# Physico-Chemical Analysis of Wastewater Discharge from Selected Paint Industries in Lagos, Nigeria

**DOI:** 10.3390/ijerph16071235

**Published:** 2019-04-07

**Authors:** Tolulope E. Aniyikaiye, Temilola Oluseyi, John O. Odiyo, Joshua N. Edokpayi

**Affiliations:** 1 School of Environmental Sciences, University of Venda, Private Bag X5050, Thohoyandou 0950, South Africa; 2 Department of Chemistry, Faculty of Sciences, University of Lagos, Akoka, Lagos 100123, Nigeria; toluseyi@unilag.edu.ng; 3 Department of Hydrology and Water Resources, University of Venda, Private Bag X5050, Thohoyandou 0950, South Africa; john.odiyo@univen.ac.za (J.O.O.); Joshua.Edokpayi@univen.ac.za (J.N.E.)

**Keywords:** wastewater treatment, paint industries, percentage efficiency, biochemical oxygen demand

## Abstract

Effluents from the paint industry have been a major source of environmental pollution. There is a need to investigate the compliance of wastewater discharged from paint industries with regulatory standards. In response, this study evaluates the physicochemical parameters of both raw and treated wastewater, the wastewater treatment plants (WWTPs) efficiencies as well as the compliance level of five selected paint manufacturing companies in Lagos, Nigeria with some regulatory standards: Federal Ministry of Environment (FME) in Nigeria, World Health Organization (WHO) and Department of Water Affairs (DWA) in South Africa. All parameters investigated were analysed using standard methods. The values of pH, electrical conductivity (EC) and total dissolved solids (TDS) levels were in the range of 4–12.2, 149.1–881.3 mS/m and 1100–6510 mg/L, respectively. The range of other parameters include total suspended solids (TSS); 0–2470 mg/L, TS; 1920–6510 mg/L, chloride; 63.8–733.8 mg/L, dissolved oxygen (DO); 0–6.7 mg/L, oil and grease (O & G); 44–100 mg/L, biochemical oxygen demand (BOD); 162.8–974.7 mg/L, chemical oxygen demand (COD); 543–1231 mg/L, nitrates;12.89–211.2 mg/L, phosphate; below detection limit (bdl)–0.02 mg/L, sulphate; 195–1434 mg/L, nickel; bdl–1.9 mg/L while copper, lead and chromium were below detection limits. The results indicated that the WWTPs of the studied paint companies were ineffective in reducing the TS, TSS, BOD, COD and (O & G) to acceptable limits. Routine monitoring of wastewater from paint industries is therefore recommended to prevent the risk of contamination to the receiving watershed which many communities rely on as source for domestic water.

## 1. Introduction

Water is an indispensable natural resource essential for the existence of man and the ecological system. Though water is abundantly available in the universe, only 3% of it is fresh water. Approximately, 5% of the fresh water, equivalent to 0.15% of the entire global waters is readily accessible for beneficial purposes [1]. In addition, water serves as an important resource for proper running of industries [2], with a majority ending up as industrial wastewater [3]. In the last few decades, anthropogenic activities coupled with rapid urbanization and industrialization have brought about ecological pressure on aquatic environment which directly or indirectly affects human health. The aquatic ecosystem often gives a reflection of extent of environmental degradation from various anthropogenic activities [4].

Water pollution plays a role in the occurrence of global ‘water crisis’, by reducing the quantity of freshwater resources available to man as well as the ecosystems. Shortage of freshwater is presently occurring in developing nations such as India, China and many African countries, as well as some developed countries [5]. Globally, 2.1 billion people are deprived of accessibility to clean water and about 4.5 billion have no access to adequate sanitation [6]. A recent UN report indicates that by 2025, two-thirds of the population of the world could face water stress. The scarcity of water could be in the form of physical scarcity, where water availability is limited and demands are not met, or it could be in the form of economic scarcity, where although water is available, there are no means/ infrastructure to provide water of required quantity and quality [3].

Industrial wastewater released into the water bodies is one of major sources of environmental pollution [7]. The discharge of wastes into the water bodies by man had brought about modification of the environmental water quality, hence making substantial quantities of water unsuitable for various uses [5]. Compromise in the quality of the environment as a result of effluent discharge from the industrial sectors has become an environmental issue for many countries especially developing nations like Nigeria [8]. In the past century, there has been rapid industrial development. This has led to an increase in the complexity of toxic effluents [9]. Release of this industrial wastewater into the environment creates a remarkable impact on the receiving water bodies. This is especially true for chemical and allied process industries like the paint industry [3].

Emulsion paints are complex mixtures composed of both organic and inorganic pigments, latexes, extenders, cellulosic and non-cellulosic thickeners, emulsifying agents, etc. [10]. Paint effluent often contains all the components of the precursor paints with insignificant dilution [11]. They could be clean-up waters composed of residual acids, plating metals and toxic chemicals [12]. The numerous chemicals used for the production of paint are responsible for the high concentrations of organic compounds, suspended solids, coloured materials and hazardous pollutants like heavy metals in the generated wastewater [10]. Some components of these wastes contain hazardous chemical elements which when discharged into the environment may penetrate and leach into the subsurface environment and subsequently settle in the soil and sediment of water bodies [13]. Heavy metals are known to be persistent and can become bioavailable for uptake by other aquatic organisms under favorable conditions. Health challenges like genetic mutation, deformation, cancer, kidney problems etc., have been linked to pollution by heavy metals [12].

It is widely known that in many low-income nations, industrial and environmental standards are non-existent, and where they are available, the mitigation instruments are inefficient. This is mainly due to lack of reliable and extensive monitoring system for industrial emissions as well as enforcement of compliance with the industrial standards [12]. Non-restricted disposal of several tones of effluents into the lagoon, rivers and streams had become a treat to the aquatic environment. Olaniyi et al. [14] revealed that discharges of untreated or partially treated wastes composed of algal nutrients, non-decomposable organics, heavy metals and other toxicants will result in compromise in quality of the receiving water bodies. The released wastewater get distributed in the soil, destroying some micofauna, hence hindering the biodiversity of microbial organisms in the soil [7]. Treatment of wastewater prior to discharge into the environment is therefore essential to prevent pollution [14].

Lagos is bounded by several bordering water bodies. Due to inadequate supply of drinkable water, water from some neighbouring rivers are used for domestic purposes. The host communities also depend on the water bodies for their means of livelihood. The water bodies in Lagos are used for activities such as fish farming, irrigation, recreation, transportation, cooling, etc. In order to protect the lives of humans, aquatic animals as well as plants relying on these water bodies for survival, it is crucial to ascertain the quality and compliance level of the released industrial wastewater with the relevant regulatory standards; Federal Ministry of Environment (FME) [15], World Health Organisation (WHO) [16] and Department of Water Affairs (DWA) [17]. Hence, this study assesses both the raw and treated wastewater qualities of five selected paint industries in Lagos, Nigeria.

## 2. Experimental Section

### 2.1. Description of Study Area

**Figure 1:**
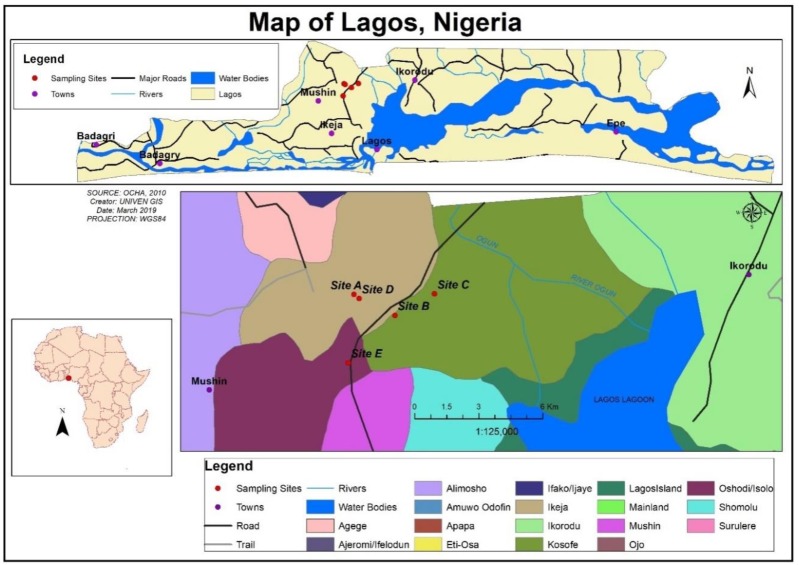
Map showing the study area.

The study was conducted in Lagos. Lagos is the smallest state of Nigeria, located in the south-western region of the country. Lagos is situated at Latitude 6°27′14″ N and Longitude 3°23′40″ E (Figure 1) with an elevation of 11 meters above sea level. Lagos has a total land mass of approximately 3577 square kilometres, with inclusive water bodies. It shares common boundaries with Ogun State at the north and east, and it is bounded at the south and west by the Coast of the Atlantic Ocean and the Republic of Benin, respectively. The vegetation in Lagos is predominantly tropical swamp forest which comprises of fresh water and mangrove swamp forests. The state experiences two major seasons yearly, which include wet season (April to October) and the dry season (November to March). The average atmospheric temperature ranges between 30 °C and 38 °C. Bulk of the water supply in Lagos comes from the Lagos and Lekki Lagoons, Atlantic Ocean, Yewa and Ogun Rivers [15]. Based on World Population Review of 2019 [18] statistical report, Lagos is believed to have a population of about 17.5 million. The population explosion had been adduced to industrialization coupled with other factors like urbanization, mass transportation and telecommunication revolutions [19].

### 2.2. Sample Collection

Both raw and treated wastewater samples were obtained from five selected paint companies in Lagos, Nigeria. The samples were collected in clean 1 L plastic bottles which had been carefully rinsed with the respective wastewater samples prior collection and labelled and were labeled as A, B, C, D and E. Onsite measurement of pH was carried out using a pH meter. The samples were transported in ice chest to the laboratory and preserved in the refrigerator prior to analyses.

### 2.3. Chemical Oxygen Demand (COD)

Determination of COD in water mainly involves the reaction of the water sample with strong oxidising agent which oxidizes the organic matter in it. COD of the wastewater sample was obtained through open reflux method. This was carried out by the addition of mercuric sulphate and sulphuric acid into an aliquot of wastewater sample in a reflux flask. On cooling, the obtained solution was reacted with known concentration of potassium dichromate and known volume of sulphuric acid. The solution was refluxed for 2 h and cooled. The obtained solution was diluted to twice its volume, cooled to room temperature and excess K_2_Cr_2_O_7_ in it determined by titrating with ferrous ammonium sulphate (FAS) using ferroin indicator. Similarly, a blank with all reagents added to 25 mL of distilled water was titrated:

(1) $$COD\;\left(mg\;L^{-1}\right)=\;\frac{\left(A-B\right)\times C\;\times 8000}{Volume\;of\;the\;sample\;\left(mL\right)}$$

where *A* = volume of titrant used for the sample (mL); *B* = volume of titrant used for the blank sample (mL); *C* = the normality of the ferrous ammonium sulphate.

### 2.4. Dissolved Oxygen (DO) Measurement

DO in the collected wastewater samples were determined with Winkler’s method. The Winkler method involves ‘trapping’ the DO in the water sample by reacting it with series of reagents resulting in the formation of an acid compound in the presence of iodine. The iodide solution was then titrated with an appropriate neutralising reagent. The change in colour signifying the end point is equivalent to the quantity of DO in the water sample [20].

### 2.5. Biochemical Oxygen Demand (BOD)

BOD in water is basically determined by the difference in the dissolved oxygen (DO) levels of water samples prior incubation and after 5 days of incubation. The BOD of the collected wastewater samples was determined by the dilution method. Dilution water was prepared by addition of 10 mL of each of the reagents; phosphate buffer, magnesium sulphate, calcium chloride, ferric chloride, sodium sulphite and ammonium chloride into 10 L of water. A measured volume of wastewater sample was topped up with dilution water to 1 L mark of a standard flask. Two 300 mL amber bottle were completely filled with the diluted water. One of the bottles was incubated at 20 °C for 5 days. MnSO_4_ solution, alkali-iodide-azide reagent and concentrated sulphuric acid were added into the other amber bottle. DO in the wastewater sample was derived through iodometric titration. For dissolved oxygen at day zero (DO_0_), 50 mL aliquot of the solution was titrated against sodium thiosulphate solution using starch solution as indicator, until a colourless end-point was attained. At the end of the 5 days, the sample in the incubator was brought out, dissolved oxygen at day five after incubation (DO_5_) was determined by following the same procedure used for the determination of DO_0_. A blank was prepared in a transparent bottle for DO_0_. Another blank was prepared in an amber bottle and incubated with the sample for DO_5_:

(2) $$BOD_{5}\left(mg\;L^{-1}\right)=\frac{\left(DO_{0}-DO_{5}\right)\;\times Volume\;of\;BOD\;bottle}{Volume\;of\;sample}$$

### 2.6. Oil and Grease (O& G) Determination

A partition gravimetric method was used to quantify the oil and grease in the wastewater samples. This was carried out through liquid-liquid extraction technique with hexane serving as the extracting liquid. An aliquot of wastewater sample was poured into a separating funnel and 25 mL hexane added. Two immiscible liquids layers were obtained with hexane forming the upper layer. The aqueous layer was collected through the tap of the separating funnel while the organic phase (hexane) was poured into a conical flask. The sample was sequentially extracted with three aliquots of hexane in the separating funnel. The solvent extracts were collected together and evaporated to dryness at ambient temperature (20–25 °C) in a fume cupboard. The difference in weight is equivalent to oil and grease in the sample:

(3) $$Oil\;and\;Grease\;\left(mg\;L^{-1}\right)=\;\frac{A-B\;\left(mg\right)\;\times \;1000}{Volume\;of\;sample\;\left(mL\right)}$$

where *A* = Total gain in weight for experimental sample (mg); *B* = Gain in weight for blank (mg).

### 2.7. Nitrate Determination

Nitrate in wastewater was determined using a UV spectrometric method. A 100 mg/L standard solution of nitrate was made by dissolving 0.72 g of anhydrous potassium nitrate in 1 L of distilled water. Serial dilutions from nitrate stock solution was done for the preparation of calibration standards for nitrate in the range 0.1–1.0 mg/L. A series of reaction tubes was set up in test tube stand and placed in a cold-water bath. Measured volume of wastewater sample was poured into the reaction tubes, with NaCl solution and sulphuric acid added sequentially. Brucine-sulphanilic acid reagent was added and the mixture heated for some minutes in a boiling water bath. The samples were then allowed to cool and the absorbance of each sample at 410 nm in the UV spectrometer was measured, in comparison with the reagent blank. Nitrate- Nitrogen (NO_3_-N) concentration in the wastewater samples was determined by extrapolation from the calibration curve.

### 2.8. Phosphate Determination

Phosphate level in the water was determined by UV spectrophotometric method. 20 mg/L of phosphate standard solution was made by dissolving 0.877 g of potassium dihydrogen phosphate in 800 mL of distilled water and making up the solution to 1 L. For the detection of phosphate, conditional reagents were prepared by mixing appropriate quantities of sulphuric acid, potassium antimonyl tartrate solution, ammonium molybdate solution and diluted ascorbic acid solution. Dilute sulphuric acid and some conditional reagent were added to a known quantity of wastewater sample using phenolphthalein as indicator. The absorbance of each sample at 880 nm was measured, using reagent blank as the reference solution. A calibration curve was plotted taking various concentrations of the standard phosphate solution with specified amount of conditional reagents.

### 2.9. Sulphate Determination

Sulphate concentration in the wastewater was determined by spectrophotometric method. Conditional reagents were prepared by mixing appropriate amount of chloride compound, alcohol, concentrated acid and distilled water. 100 mg/L of standard sulphate solution was prepared by dissolving 4.438 g of anhydrous sodium sulphate in 500 mL of distilled water and diluting the solution to 1 Series of standards, blank and known volume of the wastewater sample were prepared separately in flat bottom flasks. 5 mL of the conditioning reagent was added to each of the flat bottom flask and topped up to 100 mL after which 10 mg of barium chloride was added. The solutions became turbid and were then measured with a UV-Visible spectrometer at 420 nm. The sulphate concentration in the wastewater sample was determined with reference to the graphical representation obtained for the standard solutions.

### 2.10. Total Metal Analysis

Wastewater samples were digested by nitric acid and the digest were analyzed using Atomic Absorption Spectrophotometry (AAS, Perkin—Elmer, Melville, NY, USA). Calibration curves were plotted for each of the metals separately, by running various concentrations of standard solutions at specified wavelengths. A reagent blank sample was also analysed. The concentration of the metal was obtained from the difference between the readings of the samples and that of the blank.

### 2.11. Electrical Conductivity (EC)

The EC of the raw and treated wastewater samples for the selected five paint companies were carried out with aid of a salinometer.

### 2.12. Determination of Total Dissolved Solids (TDS)

TDS in the wastewater sample was quantified by gravimetric method. A clean Petri dish was subjected to a temperature of 100 °C in an oven, cooled in a desiccator and then weighed to constant weight. The collected wastewater sample was filtered into a clean conical flask using a pre-weighed filter paper. A known volume of the filtrate was poured into the petri-dish and heated in an oven at temperature 180 °C. The obtained residue was then cooled in the desiccator and weighed to a constant weight. The TDS is calculated with the formula below:

(4) $$Total\;Dissolved\;solids\;\left(mg\;L^{-1}\right)=\frac{\left(A-B\right)\;\times \;1000}{Volume\;of\;Sample\;\left(mL\right)}$$

where *A* = weight of dried residue + evaporating dish (mg); *B* = weight of the evaporating dish (mg).

### 2.13. Determination of Total Suspended Solids (TSS)

TSS level in the collected water samples was determined using gravimetric method. A homogeneous aliquot of water sample was filtered through a pre-weighed glass fiber filter. The filter was dried in the oven at temperature of 105 °C overnight. The filter paper was removed and allowed to cool to room temperature in a desiccator and weighted to constant weight. The increase in mass of the dry filter paper was later recorded and used for calculating TSS.

TSS is calculated using the formula:

(5) $$Total\;suspended\;solids\;\left(mg\;L^{-1}\right)=\frac{\left(A-B\right)\;\times \;1000}{Volume\;of\;Sample\;\left(mL\right)}$$

where *A* = weight of the filter after filtration (mg); *B* = weight of the filter before filtration (mg).

### 2.14. Determination of Total Solids (TS)

TS concentration in water is determined by gravimetric method. An aliquot of water sample was poured in a pre-weighed Petri dish and subjected to heat in an oven at 180 °C. The residue was then cooled in the desiccator and weighed to a constant weight.

TS is calculated with the formula:

(6) $$Total\;solids\;\left(mg\;L^{-1}\right)=\frac{\left(A-B\right)\;\times \;1000}{Volume\;of\;Sample\;\left(mL\right)}$$

TS could also be determined by the addition of TSS and TDS

(7) $$Total\;Solids\;\left(mg\;L^{-1}\right)=Total\;Susupended\;Solids+Total\;Dissolved\;Solids$$

where *A* = weight of dried residue + evaporating dish (mg); *B* = weight of the evaporating dish (mg).

### 2.15. Chloride Determination

The concentration of chloride ions in the wastewater was determined using Mohr’s method. The Mohr method uses chromate ions as an indicator in the titration of chloride ions with a silver nitrate standard solution. A known volume of wastewater sample was titrated against a known concentration of silver nitrate. After all the chloride has been precipitated as white silver chloride, the first excess of titrant resulted in the formation of a brownish red silver chromate precipitate, indicating the end point. The reactions are:

(8) $$Ag^{+}+Cl^{-}↔AgCl$$

(9) $$2Ag^{+}+CrO_{4}^{2-}↔Ag_{2}CrO_{4}$$

The concentration of chloride in the wastewater was determined from the stoichiometry and moles consumed at the end point.

### 2.16. Compliance Study and Calculation of Percentage Reduction Efficiencies

Federal Ministry of Environment in Nigeria (FME [15]) and World Health Organisation (WHO [16]) and Department of Water Affairs (DWA [17]) guidelines were used as yardsticks to assess the satisfactoriness of the effluent from the WWTP due to the prevailing environmental conditions in Nigeria and the scope of parameters stated in the guidelines. The reduction efficiencies of the various parameters were calculated by equation below:

(10) $$Reduction\;efficiency=\;\frac{Concentration\;in\;the\;influent\;-\;Concentration\;in\;the\;effluent}{Concentration\;in\;the\;influent}\;\times \;100$$

## 3. Results and Discussion

### 3.1. Chemical Oxygen Demand (COD)

**Figure 2:**
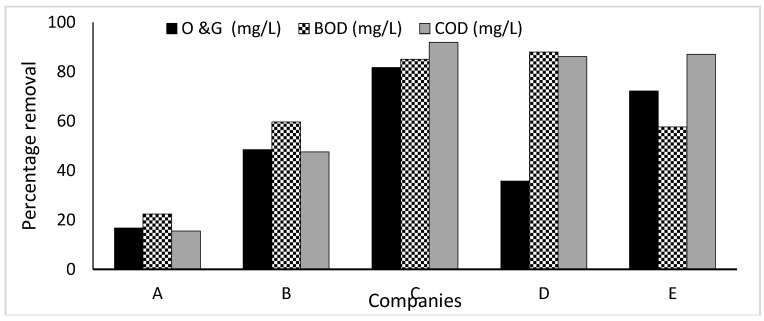
Wastewater Treatment Plant (WWTP) removal efficiencies of Companies A, B, C, D and E for Oil and Gas (O & G), Biochemical Oxygen Demand (BOD) and Chemical Oxygen Demand (COD).

Chemical Oxygen Demand is the measure of oxygen equivalent of the organic content of the sample that is susceptible to oxidation by a strong chemical oxidant. It is an evaluation used to measure the level of water contamination by organic matter [21]. The COD value is usually higher than the BOD because some organic materials in the water that are resistant to microbial oxidation and hence not involved in BOD could be easily chemically oxidized. COD measurements can be made in a few hours while BOD measurements usually take five days (BOD_5_) [22]. The COD of the raw wastewater generated from companies A, B, C, D and E were 1101 mg/L, 1198 mg/L, 6662 mg/L, 9412 mg/L and 9481 mg/L, respectively. Results obtained after treatment gave 15.5%, 47.5%, 91.85%, 88% and 87% reduction efficiencies in COD levels of WWTPs used in Companies A, B, C, D and E, respectively (Figure 2). However, despite these reduction efficiencies, the resulting COD values greatly exceeded the limits for effluent discharge of 30, 75 and 150 mg/L, respectively [15,16,17]. The presence of oxidizable inorganic compounds like extenders, pigments and additives were accountable for the high concentration of chemical oxygen demand in the samples. This inability of the industries to reduce the concentration of COD levels in WWTP below guideline value is a cause for concern among paint industries in Lagos, Nigeria.

The use of low- cost adsorbent such as activated rice husk and activated date pits in WWTPs has been found to bring about 83% and 76% COD removal efficiency respectively [23]. Hence, the use of these cost effective adsorbent aid in minimising damage to the aquatic ecosystem while still maximising profit for the paint industry. Another cost effective technology for COD reduction is the use of ultraviolet (UV) light for wastewater treatment, which showed COD removal efficiencies of 94% to 99% [24]. This high efficiency range was attributed to the photolysis of organic compounds in the wastewater by UV light as well as its capability to catalyse the release of hydroxyl ions. Furthermore, several studies in developing countries have revealed the ineffectiveness of paint industry’s WWTP in reducing the COD levels to comply with regulatory standards [22,25,26]. The resulting impact of releasing such effluent on fresh water course is significant.

### 3.2. Dissolved Oxygen (DO)

Dissolved oxygen is essential for the survival of aquatic life, and thus it serves as an important indicator of ecosystem condition [27]. DO levels in water is partly dependent on the chemical, physical and biochemical activities occurring in the water [28]. Dissolved oxygen concentrations are directly dependent on oxygen generation through photosynthesis and consumption by living organisms especially bacteria [28]. In addition, dissolved oxygen is influenced by water temperature, water movement and salinity among others [27]. Oxygen has a limited solubility in water directly related to atmospheric pressure and inversely related to water temperature and salinity [28]. Prior to treatment, the DO level of raw wastewater from the five selected industries were 0. However, the treatment of the influent in companies A, B and C resulted in the improvement of the DO to 1.1, 1.6 and 6.7, respectively (Table 1). DO level of company B even after treatment remained at 0, this showed that the WWTP is not efficient for the improvement of DO. With respect to FME [15] guideline value of 4 mg/L, all the selected companies except C are non-compliant, however based on WHO [16] standard of 1 mg/L, only treated effluents from company D and E with DO concentration of 0 mg/L do not fall within the specification for effluent discharge into the surface water. Low DO concentration in effluent is an indication of high microbial activities in the water due to presence of biodegradable organic compounds like styrene acrylate binder, cellulosic thickener, etc. in the effluent. Excessive nutrient loading can lead to depletion of dissolved oxygen by stimulating algae bloom consequently suffocation and death of aquatic organisms [27], therefore effluent from company D and E are unsafe for discharge into the waterbodies.

### 3.3. Biochemical Oxygen Demand (BOD)

BOD is the amount of oxygen utilised by microbial organisms to decompose organic compounds in water. BOD test is used to determine the extent of pollution of a wastewater and the efficacy of effluent treatment methods. DO is greatly influenced by the BOD level in water. The higher the BOD concentration, the greater the extent of oxygen depletion in the water bodies. This results in the reduction of oxygen available for higher forms of aquatic life which consequently leads to the death of aquatic organisms [29]. The treatment of the influents brought about 22.4%, 59.6%, 85%, 35.7% and 57.7% reduction of the BOD in companies A, B, C, D and E, respectively (Figure 2). The effluents from all the selected companies with BOD values of 840.6, 502.9, 162.8, 974.7 and 595.8 mg/L did not comply with FME [15] (6 mg/L) and WHO [16] (60 mg/L) BOD standards. High BOD level in the effluent could be attributed to the availability of organic compounds such as pure acrylic and styrene acrylic binders, cellulose thickener and organic pigments which could be broken down by micro-organisms. Microbial activities in the effluent results in the depletion of DO in the wastewater. Low BOD levels in the wastewater sample from company C corroborates with the high DO concentrations. Similar BOD values of 535.8, 600 and 828 mg/L were reported in India [22], Ethiopia [25] and Morocco [30]. In addition, BOD range of 3853–4691 mg/L was reported foreffluent from a textile industry in India [7]. The consequences of high BOD are similar to those for low dissolved oxygen; aquatic organisms become stressed, suffocate and die. The discharge of wastewater with high levels of BOD into waterbodies can cause serious dissolved oxygen depletion and death of aquatic animals in the receiving water bodies.

BOD and COD often give indications of the extent of organic pollution in water andwastewater [31]. BOD/COD ratio of wastewater is referred to as the biodegradability index, usually used to estimate the likelihood of organic components degradation in wastewater prior to treatment. Wastewater with BOD/COD value greater than 0.6 is considered fairly biodegradable and could be effectively treated biologically [32]. The high BOD/COD values of 1 obtained for raw wastewater from companies A and B were indications of the presence of high level of easily biodegradable compounds. However, on treating the waste water, the BOD/COD values of the resultant effluents of these two companies reduced but were still above 0.6 (Table 1). This showed that treatement of waste water through biological means would have being a more suitable method over the chemical method used by these two paints companies. The low BOD/COD values of 0.2, 0.2 and 0.2 recorded for companies C, D and E showed chemical treatment as the more preferred method of treatment over the biological means. For the sustainability of aquatic organisms, it is essential for the surface water to have reduced level of BOD/COD values. The DO concentration in water is inversely proportional to BOD/COD level [31]. This implies that low BOD/COD is associated with high DO which is the case of effluent from paint company C with BOD/COD and DO levels of 0.3 and 6.7. In contrast, effluent from companies A, B, D and E had high BOD/COD ratio which correlated with their respective low DO concentrations (Table 1).

### 3.4. Oil and Grease (O & G)

Oil and grease are highly viscous gelatinous lubricants that float on water due to their low density. The presence of high level of O and G in the water bodies can reduce productivity in the water [33]. The treatment of paint influents in the understudied paint companies had brought about 16.7%, 48.5%, 81.7%, 80.1% and 72.2% reduction efficiencies in O and G levels of WWTP used in companies A, B, C, D and E respectively (Figure 2). Although WHO [16] do not have standard for O and G, based on FME [15] and DWA [17] wastewater discharge limits of 0.1 mg/L and 2.5 mg/L respectively, all of the analysed wastewater samples greatly exceeded the required regulatory limits and therefore unsafe for disposal. High oil and grease concentration in effluent discharged into water bodies contributes to the emigration and death of aquatic animals. In addition, oil and grease on the surface of the water prevents the penetration of sunlight necessary for the photosynthetic activities of aquatic flora consequently reducing oxygen concentration in the water bodies [33].

### 3.5. Nitrates and Phosphate Removal

**Figure 3:**
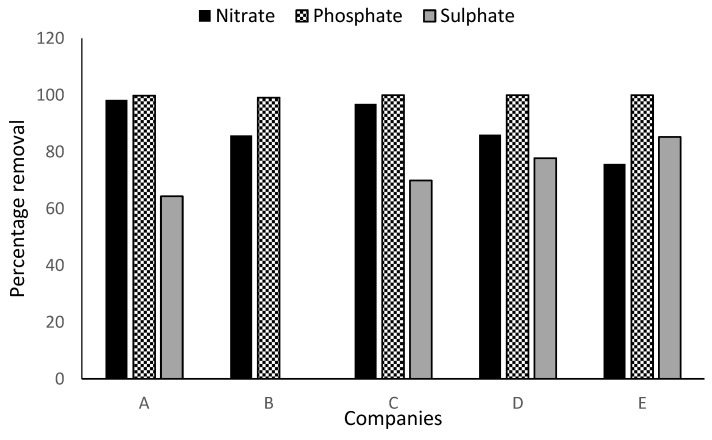
WWTP removal efficiencies of Companies A, B, C, D and E for Nitrate, phosphate and sulphate.

Nitrates are the end product of the aerobic decomposition of organic nitrogenous matter [34] while phosphate is an essential nutrient for plants [29]. Nitrate and phosphate are especially important in water pollution because they are effective nutrient sources for algae (organic nitrogen converted to ammonia, then ammonia is oxidized to nitrate, and organic phosphorus converted to inorganic phosphate) [29]. Excessive presence of phosphate in conjunction with nitrates and potassium, causes algal blooms which result in the death of aquatic organisms [29]. Nitrate reduction efficiencies of 98.3%, 85.8%, 96.9%, 86.2% and 75.8% were recorded for companies A–E, respectively (Figure 3). However, only effluent from company B met the effluent discharge regulatory standards; FME [15], WHO [16] and DWA [17] limit of 40, 45 and 15 mg/L, respectively. Companies A and C’s effluent met FME [15] and WHO [16] specifications but slightly exceeded DWA [17] requirement for nitrate disposal. Despite the high removal efficiency of 86.2% and 75.8% recorded for company D and E, their WWTPs were ineffective in the reduction of the nitrate to acceptable levels. Availability of nitrogenous compounds such as nitrocellulose resin and thickener in the effluent could be responsible for the high level of nitrate in effluent from company D and E. The discharge of such effluents can lead to nutrient loading of the receiving watershed resulting in eutrophication. Conversely, the phosphate level of raw wastewater from company A–E were 13.4, 2.3, 7.3, 6.5 and 10.3 mg/L respectively. On treatment, the phosphate in the wastewater samples from all the understudied companies were reduced to negligible levels which fell within FME [15], WHO [16] and DWA [17] limit of 3.5, 15 and 10 mg/L, respectively.

### 3.6. Sulphate

Sulphate is an essential nutrient for tissue growth in plants and animals [35]. The reduction-oxidation ability of sulphates via chemical and microbiological pathways, makes them an important link in the global sulphur cycle. The reduction of sulphates by microorganisms resulting in the linkage of sulphur and carbon biogeochemical cycles is an essential mechanism for the break-down of marine benthic sediments in anoxic state [34]. On treatment, the obtained sulphate reduction efficiencies were 64.4%, 69.9%, 77.8% and 85.2% for companies A, C, D and E, respectively. However, treatment of raw wastewater from company B resulted in an increase in sulphate level from 72.8 to 1434 mg/L. As shown in Figure 3, WWTP used in all the examined paint companies except company B were efficient in the reduction of sulphate. Based on the FME [15]/ WHO [16] guideline value of 500 and 300 mg/ L respectively, effluents from companies C, D and E fell within the specifications while effluents from company A only complied with FME [15] standard. However, effluent of company B do not comply with any of these standards. Release of effluents with high sulphate concentration can boost the release of metals from streambed sediments, resulting in increased stream alkalinity, which can adversely affect aquatic organisms having low tolerance level for high pH [35].

### 3.7. Heavy Metals

Heavy metals are potentially toxic trace elements, and their impacts may be felt in organisms at low concentrations [36]. Heavy metals are not biodegradable, hence tend to bio- accumulate in aquatic organisms [37]. Heavy metals such as cadmium, mercury, nickel, zinc, chromium, copper among others are toxic at high concentrations. The oral route has been detected as the main means through which heavy metals get into the human body system. People who fed on farm produce irrigated with untreated and partially treated wastewater are prone to various ailments which effect might not be immediate [31]. The concentration of nickel in both the raw and treated effluent from all the examined companies except A and B were below detection limit (Table 2). 78% reduction efficiency was observed in WWTP used by company A, however the treatment of wastewater generated by company B brought about no reduction in the nickel concentration of 1.9 mg/L. In essence, all the selected paint companies except A and B meet the FME [15] and WHO [16] acceptable requirement of 0.1 and 0.2 mg/ L for nickel, respectively. Toxic level of nickel in water bodies can cause modification of immunological response of aquatic animals resulting in physiological changes in them [38]. Haematotoxic effects as well as growth retardation were reported by Oladele et al. [39] in swiss albino mice on their exposure to paint effluents having high concentrations of metals and dissolved ions. The presence of nickel in the aquatic ecosystem can also cause weight loss, fork length increments as well as reduction in feed conversion efficiency (FCE) of aquatic animals [40]. In addition, chromium, copper and lead were found below detection limits in both the raw and treated wastewater of all the understudied paint companies.

### 3.8. Chloride

For safety reasons, chloride in wastewater should not exceed 350 mg/L as directed by FME [15] and WHO Standard [16]. Prior treatment, the chloride value of the understudied paint companies was 159.5, 159.5, 95.7, 287.1 and 127.6 mg/L for companies A, B, C, D and E, respectively (Table 2). However, on treatment, all the companies’ effluents except Company B and C fell within the acceptable regulatory limits. In water bodies, elevated chloride levels can threaten the sustainability of ecological food sources, hence posing a risk to species survival, growth as well as reproduction [41]. Chloride removal efficiencies for WWTPs used by companies A, D and E were 60%, 55.6% and 25%, respectively. However, increase in chloride level were recorded for company B and C. The high chloride level in effluents from company B and C could be attributable to the presence of chlorinated rubber resin, chlorinated solvents like methylene chloride, cationic surfactants like trimethyl octadecyl ammonium chloride and alkyl benzyl ammonium chloride as well as polyvinyl chloride present in the parent paint. Bio-accumulation and persistence of chloride may affect aquatic organisms and water quality [35].

### 3.9. Electrical Conductivity (EC)

EC is the measure of a solution’s ability to conduct electric current which is greatly dependent on the availability of ionic species [28]. Inorganic ions have major influence on the conductivity of water. High values of EC show that inorganic ions are in abundance in the wastewater. EC is directly proportional to the total dissolve solids (TDS) concentration. In essence, high EC in wastewater is an indication of high total dissolved solids concentration. This also implies that the ability of an electric current to pass through the wastewater is proportional to the concentration of ionic solutes dissolved in the water [42]. Treatment of wastewater from all the selected companies resulted in increase in EC from 122 to 149.1 mS/m, 54.2 to 311.8 mS/m, 108.5 to 881.3 mS/m and 95 to 149.2 mS/m, for A, B, C and E respectively (Table 3). However, reduction in EC from 298.4 to 162.8 mS/m was observed in wastewater from Company D. This shows that the WWTP of company D is 45.4% efficient in the reduction of EC even though DWA [17] effluent discharge limit of 70–150 mS/m was not met. The result is comparable with the EC range of 435.4–576.8 mS/m reported for discharge wastewater from textile company that uses large quantities of paints in India [7].The sporadic rise in EC observed in all the WWTPs except that for D could be due to the presence of dissolved ions of resins, thinning agents and additives used in the production of the parent paint coupled with dissolved ions of the reagents used for the wastewater treatment. The effluent discharge from Companies A and E WWTP met the DWA [18] specification. The inability of the EC of the effluent from company B and C to comply with regulatory limit, showed that reagents were excessively used for the treatment of the effluent, hence the effluent is not suitable for discharge into the surrounding water bodies. Discharge of wastewater with high EC into the surrounding watershed may bring about water imbalance for aquatic organisms and could greatly decrease dissolved oxygen concentration [41].

### 3.10. pH

The pH of wastewater during treatment is essential for the removal of organic compounds and heavy metals. Alkaline pH favours the precipitation of most metals as insoluble solids [43]. The mean value of pH obtained for the raw wastewater samples was 7.33 ± 0.87 an indication that the five paint industries being examined use production procedures that ended up in the production of raw wastewater of almost the same pH. Treatment of the raw wastewater from companies A, B, C and D resulted in slight variations in pH values from 6.9 to 7.5, 6.6 to 4, 7.4 to 12.2, 8.8 to 6.9 respectively while raw wastewater of company E with pH value of 7 remained unchanged (Table 3). The pH range obtained in this study correlate with the pH range of 5 to 11 achieved in the study carried out by Eremektar et al. [44] when different types of coagulants were used for the treatment of paint effluent. The pH of treated wastewater from company B (pH 4) and C (pH 12.2) did not fall within FME [15], WHO [16] and DWA [17] effluent discharge limits 6.5–8.5, 6–9 and 5.5–9.5 respectively. The acidic nature of Company B’s effluent could be attributed to the presence of acidic compounds such as phosphoric acid used in the production of the parent paint as well as the excessive use of alum (Al_2_O_3_) or the use of inadequate amount of lime (CaO) for the raw wastewater treatment of company B.

Conversely, the alkalinity observed in influent from Company C could be as a result of the existence of basic compounds like calcium carbonate, ammonia, iron oxide and titanium dioxide found in the precursor paint coupled with the use of excessive amount of lime for the wastewater treatment. The pH of effluent is very important as it can negatively impact the receiving watershed. Acidic pH is known to favour the bioavailability of most metals in river systems with its attendant consequences. Low pH levels can encourage the solubility of heavy metals resulting in the release of metal cations into the water rather than being adsorbed into the sediment. Extremely low pH can bring about the migration of pH-tolerant algae resulting in algae blooms [45].

### 3.11. Total Dissolved Solids (TDS)

Total dissolved solid is a measure of all dissolved substances in water [42]. In other words, TDS is a measure of inorganic salts, organic matter and other dissolved materials in water. Treatment of raw wastewater from company D had brought about TDS reduction efficiency of 43.75%. However, the WWTP used in companies A, B, C and E resulted in a tremendous increase in the TDS level from 980 to 1100, 490 to 2330, 840 to 6510 and 730 to 1180 mg/L, respectively (Table 3). This increase could be as a result of the presence of dissolved ions of resins, organic and inorganic solvents and additives like surfactants, coalescing and anti-cracking agent found in paint as well as the dissolved ions of the chemical reagents used for the effluent treatment in these paint companies. This corroborates with the findings recorded for EC. Although, FME [15] and DWA [17] have no standard for TDS effluent discharge into surface water, however, for effluent to be reused for irrigation purpose, it should not exceed 1500 mg/L [16]. In addition, water is said to be unpalatable and may begin to loss its commercial and domestic worth when the TDS level exceeds 1000 mg/L. High concentration of TDS in water is responsible for excessive scaling in water pipes, boilers, heaters, and household appliances [46]. The result obtained in this study is similar to the range of TDS values of 4354–5768 and 2002–7463 mg/L reported on similar studies by Kaur et al. [7] and Ram et al. [22]. Among the understudied companies, only treated wastewater of company B and C exceeded the WHO [16] limit for effluent discharge. High concentration of TDS can result in dehydration of aquatic animals [29].

### 3.12. Total Suspended Solids (TSS)

TSS is a measure of particulate maters suspended in water. It is used to describe the extent of pollution in wastewater. In addition, TSS serves as a good indicator for the turbidity of the water [21]. The TSS values for the raw wastewater from paint companies A, B, C, D and E were 8090, 2470, 2400, 10,280 and 5070 mg/L respectively (Table 1). However, after treatment, percentage reduction efficiencies of 89.5%, 81.4%, 100%, 90.1% and 85.4% were recorded for the WWTPs of companies A–E, respectively (Table 3). This showed that the WWTPs of all companies were efficient in reducing the level of TSS but this removal was not sufficient as the TSS in the effluents from all the selected companies except C did not comply with FME [15], WHO [16] and DWA [17] wastewater discharge limit of 0.75 mg/L, 60 mg/L and 25 mg/L respectively. The high level in TSS could be as a result of the presence of inorganic particulate matters such as extenders, pigments and additives present in paint. The suspended solids in the effluent is within the typical ranges given in literature for similar industrial premises manufacturing paint and textile [7,12,22,25,44]. Individuals exposed to water with high concentrations of TSS and TDS are at risk of having cancer [7].

### 3.13. Total Solids (TS)

Solids in effluent could either be organic or inorganic in nature. Such solids may be present in dissolved or suspended form [44]. In essence, total solids are the summation of TSS and TDS. The TS reduction efficiencies for the WWTP used by companies A, B, D and E were 78.5%, 5.7%, 81.8% and 66.9%, respectively whereas on treatment, the TS level in wastewater from company C increased from 3240 to 6510 mg/L (Table 3). However, none of these efficiency level recorded for companies A, B, D and E was sufficient to bring its respective effluent to the acceptable FME [16] permissible requirement of 1500 mg/L for TS required for ground water to be used for domestic purpose. The result is comparable with the range of values of 3188–3973.7 and 2590–3923 mg/L for TS in paint effluents reported by Chidokie and Nkwakanma [12] and Ram et al. [22].

## 4. Conclusions

Around the world as nations are struggling to arrive at an effective regulatory regime to control the discharge of industrial effluents into their ecosystems, the Nigerian economy faces a double-edged sword of economic growth and eco-system collapse. The experimental data obtained reveals the high level of pollution introduced into the environment by paint industries. The research has shown that many paint industries most especially the small-scale industries, due to their inability to afford good effluent treatment procedure, do not treat their effluent before discharge into the environment while the WWTP of the few ones that could afford treatment are not efficient enough to bring about effluents that are eco-friendly. The results obtained from the five selected paint industries, greatly showed a non-compliance to various regulatory standards as most of the physicochemical parameters investigated exceeded the levels recommended for discharge. For some parameters, there were enrichment of the contaminants which further showed a failure in the treatment system. The result obtained also shows a linear correlation between percentage reduction efficiency of COD and BOD in the WWTPs of all the studied paint companies except D. Company C performed better than others based on the percentage reduction efficiencies of the WWTPs used in all the studied paint companies, while company A had the least performance. This study revealed that WWTPs of the five selected companies which are representatives of paint companies in Lagos are ineffective in removing TSS, TS, O and G, BOD and COD. Hence effluents generated by the paint industry serve as one of the major pollution sources of the water bodies. Routine monitoring of paint industries is therefore recommended to prevent the risk of contamination to the receiving watershed which many communities depends as source for domestic water.

## Figures and Tables

**Table 1:** **Dissolved Oxygen** (DO) concentration and Biochemical Oxygen Demand- Chemical Oxygen Demand Ratio (BOD/COD) of paint effluents in the study area.

**DO (mg/L)**	**Influent**	**Effluent**	**% Removal**
A	0	1.1	N/A
B	0	1.6	N/A
C	0	6.7	N/A
D	0	0	N/A
E	0	0	N/A
Federal Ministry of Environment (FME) Discharge standard	4 mg/L		
World Health Organisation (WHO) Discharge standard	1 mg/L		
**BOD/COD**	**Influent**	**Effluent**	**% Removal**
A	1.0	0.9	N/A
B	1.0	0.8	N/A
C	0.2	0.3	N/A
D	0.2	0.9	N/A
E	0.2	0.5	N/A

N/A—Note Applicable.

**Table 2:** WWTP removal efficiencies of Companies A–E for Nickel and Chloride.

**Nickel (mg/L)**	**Influent**	**Effluent**	**% Removal**
A	7.5	1.7	78
B	1.9	1.9	0
C	bdl	bdl	N/A
D	bdl	bdl	N/A
E	bdl	bdl	N/A
WHO Discharge standard	0.2 mg/L		
**Chloride**	**Influent**	**Effluent**	**% Removal**
A	159.5	63.8	0
B	159.5	733.8	60.0
C	95.7	382.9	0
D	287.1	127.6	55.6
E	127.6	95.7	25.0
FME Discharge standard	350 mg/L		
DWA Discharge standard	0.25 mg/L		
WHO Discharge standard	350 mg/L		

Bdl–Below detection limit.

**Table 3:** EC, pH, TDS, TSS and TS levels of the effluents of the study area.

**Conductivity (mS/m)**	**Influent**	**Effluent**	**% Removal**
A	122	149.1	0
B	54.2	311.8	0
C	108.5	881.3	0
D	298.4	162.8	45.4
E	95	149.2	0
DWA Discharge standard	150 mS/m		
**pH**	**Influent**	**Effluent**	**% Removal**
A	6.9	7.5	N/A
B	6.6	4	N/A
C	7.4	12.2	N/A
D	8.8	6.9	N/A
E	7	7	N/A
FME Discharge standard	6.5–8.5		
DWA Discharge standard	5.5–9.5		
WHO Discharge standard	6–9		
**TDS**	**Influent**	**Effluent**	**% Removal**
A	980	1100	0
B	490	2330	0
C	840	6510	0
D	2240	1260	43.8
E	730	1180	0
WHO Discharge standard	1500 mg/L		
**TSS**	**Influent**	**Effluent**	**% Removal**
A	8090	850	89.5
B	2470	460	81.4
C	2400	0	100
D	10,280	1020	90.1
E	5070	740	85.4
FME Discharge standard	0.75 mg/L		
DWA Discharge standard	25 mg/L		
WHO Discharge standard	60 mg/L		
**TS**	**Influent**	**Effluent**	**% Removal**
A	9070	1950	78.5
B	2960	2790	5.7
C	3240	6510	0
D	12,520	2280	81.8
E	5800	1920	66.9
FME Discharge standard	1500 mg/L		
